# Comparison of Weekly Paclitaxel Regimens in Recurrent Platinum-Resistant Ovarian Cancer: A Single Institution Retrospective Study

**DOI:** 10.3390/curroncol31080345

**Published:** 2024-08-15

**Authors:** Laurence Morin, Louis-Philippe Grenier, Nicolas Foucault, Éric Lévesque, François Fabi, Eve-Lyne Langlais, Alexandra Sebastianelli, Marianne Lavoie, Marc Lalancette, Marie Plante, Mahukpe Narcisse Ulrich Singbo, Vincent Castonguay

**Affiliations:** 1 Medical Oncology Division, Centre Hospitalier Universitaire de Québec-Université Laval, Québec, QC G1J 5B3, Canada; lamor82@ulaval.ca (L.M.); eric.levesque.med@ssss.gouv.qc.ca (É.L.); marianne.lavoie.med@ssss.gouv.qc.ca (M.L.); marc.lalancette.med@ssss.gouv.qc.ca (M.L.); 2 Medical Oncology Pharmacy Department, Centre Hospitalier Universitaire de Québec-Université Laval, Québec, QC G1J 5B3, Canada; louis-philippe.grenier@chudequebec.ca; 3 Pharmacy Department, Unité de Formation et de Recherche en Santé de Rouen, 76183 Normandie, France; nicolasfoucault96@gmail.com; 4 Radio-Oncology Division, Centre Hospitalier Universitaire de Québec-Université Laval, Québec, QC G1J 5B3, Canada; francois.fabi.med@ssss.gouv.qc.ca; 5 Gynecologic Oncology Division, Centre Hospitalier Universitaire de Québec-Université Laval, Québec, QC G1J 5B3, Canada; eve-lyne.langlais.med@ssss.gouv.qc.ca (E.-L.L.); alexandra.sebastianelli.med@ssss.gouv.qc.ca (A.S.); marie.plante.med@ssss.gouv.qc.ca (M.P.); 6 Centre de Recherche du Centre Hospitalier Universitaire de Québec-Université Laval, Québec, QC G1E 6W2, Canada; narcisse.singbo@crchudequebec.ulaval.ca

**Keywords:** paclitaxel, ovarian carcinoma, platinum-resistant

## Abstract

Weekly paclitaxel (WP) is a chemotherapeutic cornerstone in the management of patients with platinum-resistant ovarian carcinoma. Multiple WP dosing regimens have been used clinically and studied individually. However, no formal comparison of these regimens is available to provide objective guidance in clinical decision making. The primary objective of this study was to compare the cumulative dose of paclitaxel delivered using 80 mg/m^2^/week, administered using either a 3 weeks out of 4 (WP3) or a 4 weeks out of 4 (WP4) regimen. The secondary objective was to evaluate the clinical outcomes associated with both regimens, including efficacy and toxicity parameters. Our retrospective cohort comprised 149 patients harboring platinum-resistant ovarian cancer treated at the CHU de Québec from January 2012 to January 2023. WP3 and WP4 reached a similar cumulative dose (1353.7 vs. 1404.2 mg/m^2^; *p* = 0.29). No significant differences in the clinical outcomes were observed. The frequency of dose reduction was significantly higher for WP4 than WP3 (44.7% vs. 4.9%; *p* < 0.01), mainly due to treatment intolerance from toxicity (34.0% vs. 3.9%; *p* < 0.01). Our data suggest that a WP3 regimen delivers a similar cumulative dose to WP4, hence offering a better tolerability profile without compromising efficacy.

## 1. Introduction

In 2023, approximately 3100 Canadian women received a diagnosis of ovarian cancer [1]. Notwithstanding its low incidence, ovarian cancer represents the fifth leading cause of cancer-related death in this population and is the most lethal of all gynecological malignancies [1]. Survival rates are highly variable and are mainly related to the stage at diagnosis. While the 5-year pooled survival rate is 51%, at stage IV, the rate falls to 32% [2]. Moreover, owing in great part to the asymptomatic nature of the disease, approximately 50% of all ovarian cancer cases will be diagnosed at an advanced stage [2]. In almost all cases, the first line of treatment involves cytoreductive surgery combined with adjuvant chemotherapy, most frequently through the combination of platinum and taxane agents [3]. However, while primarily effective, most patients will relapse following initial treatment. Their disease will then demonstrate progressive resistance to further chemotherapeutic interventions, partly due to various molecular alterations, intratumoral heterogeneity and the emergence of resistant subpopulations [4,5]. More specifically, platinum-resistant ovarian carcinoma, which lies within a spectrum of resistance profiles, is broadly defined by the recurrence or progression within 6 months of exposure to platinum salts [6]. In patients exhibiting disease with such resistance, treatment options are limited.

There is currently no consensus on the optimal therapeutic strategy once platinum-resistance has emerged [3,7,8]. Weekly paclitaxel remains central to the available arsenal in this context, with the reported response rate oscillating between 20% and 62% [8,9,10,11,12,13,14,15]. However, chronic exposure to chemotherapeutic compounds is also thought to possess potent anti-angiogenic effects, underlining the potential benefit of metronomic dosing regimens [16]. In order to establish if the reduced frequency of treatments would translate into comparable effectiveness coupled with reduced toxicities, alternative schedules have been investigated. The 3 weeks out of 4 regimen (WP3) has been used in platinum-resistant ovarian carcinoma with good results [10,13,17]. Nonetheless, no prospective nor retrospective studies have directly compared the continuous weekly regimen (4 weeks out of 4; WP4) to WP3.

In this paper, we present retrospective data comparing the efficacy and safety of paclitaxel 80 mg/m^2^ administered as WP3 and WP4 regimens in 149 patients with platinum-resistant ovarian carcinoma treated at our center over the last ten years. Our results add to the paucity of data available to guide clinical decision making in this challenging population.

The primary endpoint is the cumulative dose of paclitaxel received for WP3 and WP4. Secondary endpoints are related to the occurrence of paclitaxel dose reduction, the mean paclitaxel dose received per week and the dose intensity administered (% of the maximum expected dose infused). Additional secondary endpoints are time to next treatment (TTNT), overall survival (OS), mean duration of treatment, CA-125 response, occurrence of treatment delays, hospitalizations, neurotoxicity and the need for blood transfusions.

Our hypothesis was that WP4 would not lead to a higher cumulative dose since it would require more dose reductions and lead to more toxicities than WP3. We transposed this hypothesis from a similar study with Bortezomib, where a weekly administration schedule demonstrated equivalent progression free survival (PFS) and OS with a better tolerability profile than bi-weekly administration [18].

No formal statistical hypothesis was made and all the available patients were included.

Our results demonstrate a similar cumulative dose for WP3 and WP4 with a comparable efficacy profile and better tolerability of WP3.

## 2. Materials and Methods

We have retrospectively studied a cohort of 149 patients treated at CHU de Quebec-Université Laval who received WP for platinum-resistant ovarian carcinoma between January 2012 and January 2023. The paclitaxel regimen choice (WP3 or WP4) was based on the clinicians’ personal preferences, patients’ comorbidities and clinical trends. Inclusion criteria included a diagnosis of ovarian, tubal or primary peritoneal metastatic or locally advanced carcinoma. The platinum resistance was defined as progressing within 6 months after platinum-compound exposure.

Exclusion criteria included patients receiving WP before 2012 or after January 2023, treatment with WP for another type of cancer or administration of less than one full cycle of treatment. Finally, if WP was administered and withheld for several weeks/months then reintroduced for progression as a new line, only the first treatment was used for analyses.

Patients were identified retrospectively through the chemotherapy prescription and administration database of the CHU de Quebec-Université Laval. All patients receiving WP were screened with the inclusion and exclusion criteria mentioned above. Retrospective data were then analyzed comparing the two groups head-to-head (WP3 vs. WP4). Patients were allocated to WP3 and WP4 in an intention-to-treat manner.

Main characteristics of the study population were compiled for each group (WP3 and WP4), such as age, ECOG Performance Status [19], tumor origin, histology, surgical cytoreduction, BRCA status, number of previous lines of treatment, taxane-free interval, Bevacizumab and PARP inhibitors (PARPi) exposition. The cumulative and mean dose administered, dose reduction, treatment delay and dose intensity were the collected dosing parameters. The toxicity parameters were hospitalizations, neurotoxicity, blood transfusions and treatment intolerance. The efficacy parameters were median OS, TTNT, duration of treatment and CA-125 response based on the Gynecologic Cancer Intergroup (GCIG) criteria.

The study was approved by the local ethical research committee of the CHU de Québec-Université Laval (2023–6722) and was conducted conforming to the Declaration of Helsinki. Data were double-anonymized.

Descriptive statistics (mean with standard deviation or median with interquartile range, or proportions) were used as appropriate. Wilcoxon Mann–Whitney, Pearson’s Chi Square and Student T-tests were used to compare groups. All tests were two-sided and the threshold of <0.05 for *p* values was considered to be statistically significant. The Kaplan–Meier method was used to estimate OS and TTNT. The analysis was conducted using the SAS^®^ 9.4 software.

## 3. Results

Of the 149 patients corresponding to the aforementioned inclusion and exclusion criteria, 102 formed the WP3 and 47 the WP4 group. When dividing the studied period in half, 42/102 patients received WP3 between 2012 and 2017, and 60/102 between 2018 and 2023. In parallel, 36/47 patients received WP4 between 2012 and 2017 and 11/47 between 2018 and 2023. The median follow-up time was 13.0 months. As presented in Table 1, the WP3 and WP4 patient populations were essentially comparable. The majority of patients were BRCA-negative, diagnosed with high-grade serous carcinoma and had good performance status (ECOG = 1) at initiation of WP. The median number of previous treatment lines was 3 in both groups. The taxane and platinum-free intervals were similar. The descriptive demographics of the studied cohorts are detailed in Table 1.

There was no difference in the delivered cumulative dose between the two treatment regimens (1353.7 vs. 1404.2 mg/m^2^; *p* = 0.29). An expected statistically significant difference in the mean weekly dose received between the two groups was observed (59.1 vs. 70.2 mg/m^2^/week; *p* < 0.01), since the 80 mg/m^2^/week in WP3 reduces to 60 mg/m^2^/week when reported on a 4-week schedule. There was also a significant difference in the dose intensity received (98.5 vs. 87.8% of planned dose respectively; *p* < 0.01). Still, there was no difference in the treatment delays (27.5 vs. 23.4%; *p* = 0.69) was noted (Table 2).

However, there was a significant difference in the dose reductions, with 4.9% of patients receiving WP3 requiring a dose reduction compared to 44.7% of patients receiving WP4 (*p* < 0.01) (Table 2). These dose reductions were mainly due to a difficulty in tolerating the treatment as per the clinician assessment (3.9 vs. 34.0%; *p* < 0.01) (Table 3). Nevertheless, when analyzed individually, there was no significant difference in hospitalizations (22.5 vs. 31.9%; *p* = 0.33), the occurrence of neurotoxicity (66.6 vs. 60%; *p* = 0.93) or the percentage of patients receiving packed red blood cells transfusion (4.9 vs. 10.6%; *p* = 0.29) (Table 3).

**Figure 1:**
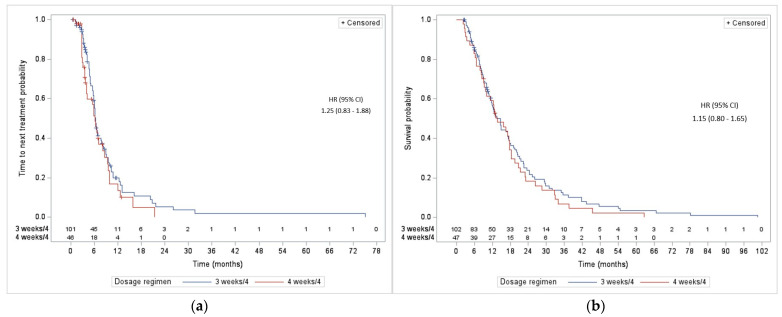
(**a**) Time to next treatment according to the paclitaxel regimen used; (**b**) overall survival according to the paclitaxel regimen used.

As demonstrated in Table 4, the median TTNT was not statistically different between both groups (6.3 vs. 6.2 months; *p* = 0.28) (Figure 1a). The median OS was also not statistically different between WP3 and WP4 (14.6 vs. 13.6 months; *p* = 0.43) (Figure 1b). The median duration of treatment was comparable in both groups with 20.4 weeks for WP3 versus 17.0 weeks for WP4 (*p* = 0.52), respectively. A 50% or more CA-125 reduction was observed in 61.5% of WP3 vs. 64.1% of WP4 (*p* = 0.79).

## 4. Discussion

The results of this retrospective analysis suggest that the administration of WP3 delivers a similar cumulative dose with a comparable efficacy to WP4, which confirms our hypothesis. The collected results did not identify a specific toxicity pattern. However, the proportion of patients requiring dose reduction with WP4 was significantly higher than with WP3, mainly due to treatment intolerance. Given the inherent limitations of the retrospective collection of the toxicity data, we hypothesize that the greater number of dose reductions is a surrogate of the increased toxicity of the WP4 regimen, even if our results do not show this explicitly in the various individually collected adverse events. As toxicity data were not collected prospectively, the retrospective assessment from the clinical notes of subjective symptoms such as fatigue, neuropathy, dyspnea, inappetence or nausea was of limited value. Nevertheless, we believe that increased toxicity is the more plausible explanation for the most frequent dose reductions with the WP4 regimen. This difference in tolerance between the two groups is also reflected in the planned dose intensity administered. Furthermore, the larger proportion of patients receiving WP4 in the first half of the time period seems to demonstrate a change in clinical practices, probably based on the observed toxicities in the WP4 group. 

When compared to pre-existing data, two European studies published in 2002 used WP4 [12,20]. The first one included 57 patients treated with WP4 for platinum-resistant disease, with a response rate of 49–56%, an OS of 13.7 months and a PFS of 4 to 5 months [12]. The second study, comparing WP4 and paclitaxel every 3 weeks in 208 patients, demonstrated a 41% response rate for WP4 with a median complete response of 4.5 months. The median response duration was 9.4 months in the WP4 group, with a time to progression of 6.1 months and a median OS of 13.6 months. It should be noted that half of the patients in this study did not have platinum-resistant tumors [20]. Lastly, a study of 37 patients from 2004 administering WP3 to platinum-refractory patients demonstrated a total response rate of 45.9% with an initial improvement of the CA-125 of 56%, using Gynecological Cancer Intergroup (GCIG) response criteria [13]. The results also showed that as the number of previous lines of treatment increases, the response to treatment decreases [13]. It should be reminded that in our study, the median number of previous lines of treatment was 3, hence our patients were more likely to be multidrug-resistant. That being said, despite the differences in the populations and outcome definitions, the performance of our WP4 and WP3 regimens seems globally similar to the literature, suggesting that the comparable results between our two groups are unlikely to be attributable to an underperformance of the WP4 cohort [12,13,20].

The strength of our study is that it is, to our knowledge, the first retrospective trial to directly address the question of the optimal WP administration regimen in platinum-resistant ovarian carcinoma. Although an indirect analysis of phase II studies or a control arm of phase III studies [8,9,10,11,12,13,14,15] may inform us of the efficacy and toxicity of these two regimens, population and study design differences make inter-study comparisons biased and thus potentially less useful than the data presented in our study.

There are limitations to our study that need to be recognized, including mainly the retrospective nature of data collection. As discussed above, we believed that it specifically impacted the exhaustivity of the collected toxicity details. In addition, there may have been biases in the clinicians’ selection of the chemotherapy regimen. However, it is probable that the frailest patients would have been assigned to the lowest dose-dense regimen (WP3), which is reassuring for the validity of the results observed. Also, the choice of a weekly, more tolerated regimen may result from frailty but also depends on the degree of toxicity of the previous treatment. Lastly, the use of growth factor was not collected, which is also a limitation that could influence the results in both groups. 

Since the WP4 regimen did not lead to a greater cumulative dose administered compared to WP3, the addition of a therapeutic break without compromising the effectiveness of treatment in terms of TTNT, OS, CA-125 response and the total duration of treatment is clinically appealing. This monthly week off can potentially free up chemotherapy chair time, which is a major issue in the context of human resource shortages in North America. This could inform institutional decisions to provide wiser resource utilization. In addition, this pause offered with WP3 may be attractive for patients with a chronic and incurable disease who often seek ways to optimize their quality of life by alleviating treatment burden and toxicity. In addition, it was estimated that the cost of one week of WP at the CHU de Québec was about CAD 252, including the nursing staff, pharmacist and medication fees. Even if not transposable to other centers due to local variations, this could potentially lead to a substantial financial advantage for the healthcare system.

## 5. Conclusions

In summary, our data support a comparable cumulative dose administered for a similar efficacy between WP3 and WP4 regimens as well as a possible advantage with regard to the toxicity profile of WP3, as implicitly reflected by a lower proportion of dose reductions in this group.

## Figures and Tables

**Table 1:** Main characteristics of the study population.

Variables	WP3 (*n* = 102)	WP4 (*n* = 47)	*p*
**Treatment period**			<0.01
2012–2017	42	36	
2018–2023	60	11	
**Age (years)**	69.0	64.0	0.06
**ECOG (%)**			0.47
0–1	87 (85.3)	44 (93.6)	
2–3	15 (14.7)	3 (6.4)	
**Tumor origin (%)**			0.18
Ovary	85 (83.3)	33 (70.2)	
Peritoneum	5 (4.9)	7 (14.9)	
Tubal	12 (11.8)	7 (14.9)	
**Histology (%)**			0.05
High-grade serous	91 (89.2)	36 (76.6)	
Others	11 (10.8)	11 (33.4)	
**Surgical cytoreduction (%)**			0.02
None	19 (18.6)	2 (4.3)	
Suboptimal	20 (19.6)	16 (34.0)	
Optimal	62 (60.8)	27 (57.4)	
Non-specified	1 (1.0)	2 (4.3)	
**Germinal BRCA status (%)**			0.42
Negative	75 (73.5)	29 (61.7)	
BRCA1	3 (2.9)	1 (2.1)	
BRCA2	2 (2.0)	2 (4.3)	
Unknown	22 (21.6)	15 (31.9)	
**#** **previous lines of treatments (%)**			0.40
1	7 (6.9)	1 (2.1)	
2	25 (24.5)	11 (23.4)	
3	40 (39.2)	14 (29.8)	
4	17 (16.7)	15 (31.9)	
5	8 (7.8)	4 (8.5)	
6	1 (1.0)	1 (2.1)	
7	4 (3.9)	1 (2.1)	
**Taxane-free interval (months)**	17.0	18.5	0.96
**Platinum-free interval (months)** **Bevacizumab (%)**	8.0 10 (9.8)	8.0 5 (10.6)	0.94 1.0
**PARPi (%)**	17 (16.7)	6 (12.8)	0.75

Regimens were administered using weekly paclitaxel either 3 weeks out of 4 (WP3) or 4 weeks out of 4 (WP4). Maximal dosage regimens were WP3 = 60 mg/m^2^/week (80 mg/m^2^/week 3 weeks out of 4, thus 60 mg/m^2^ when distributed on a 4-week schedule) and WP4 = 80 mg/m^2^/week. The percentage represents proportion of patients presenting the specified characteristics within their respective groups. Age, taxane-free and platinum-free intervals are presented as medians. Bevacizumab and PARPi represent the percentage of patients with a prior exposure to these drugs.

**Table 2:** Comparison of WP3 and WP4 dosing parameters.

Variables	WP3	WP4	*p*
Cumulative dose (mg/m^2^)	1353.7	1404.2	0.29
At least one dose reduction (%)	4.9	44.7	<0.01
At least one treatment delay (%)	27.5	23.4	0.69
Mean dose (mg/m^2^/week)	59.1	70.2	<0.01
Dose intensity (%)	98.5	87.8	<0.01

Maximal dosage regimens were WP3 = 60 mg/m^2^/week and WP4 = 80 mg/m^2^/week. Cumulative dose is expressed as a mean. Percentage for dose reduction and treatment delay represents proportion of patients who required these interventions. Dose intensity is expressed as percentage of maximal dose received.

**Table 3:** Comparison of WP3 and WP4 toxicity parameters.

Variables	WP3	WP4	*p*
Hospitalizations (%)	22.5	31.9	0.33
Neurotoxicity (%)	66.6	66.0	0.93
Blood transfusions (%)	4.9	10.6	0.29
Toxicity-related dose reductions (%)	3.9	34.0	<0.01

The percentage represents the proportion of patients experiencing the adverse effect. Toxicity-related dose reduction is defined as any treatment intolerance causing a reduction in the administered dose.

**Table 4:** Comparison of WP3 and WP4 efficacy parameters.

Variables	WP3	WP4	*p*
Overall survival (months)	14.6	13.6	0.43
Time to next treatment (months)	6.3	6.2	0.28
Duration of treatment (weeks)	20.4	17.0	0.52
CA-125 > 50% response (%)	61.5	64.1	0.79

Overall survival, time to next treatment and duration of treatment are presented as medians. CA-125 response is based on the Gynecologic Cancer Intergroup (GCIG) criteria.

## Data Availability

The raw data supporting the conclusions of this article will be made available by the authors on request.
